# A prognostic nomogram for T3N0M0 esophageal squamous cell carcinoma patients undergoing radical surgery based on computed tomography radiomics and inflammatory nutritional biomarkers

**DOI:** 10.1002/acm2.14504

**Published:** 2024-09-06

**Authors:** Hui Ma, Yangchen Liu, Hongxun Ye, Fei Gao, Songbing Qin

**Affiliations:** ^1^ Department of Radiation Oncology The First Affiliated Hospital of Soochow University Su Zhou Jiangsu Province People's Republic of China; ^2^ Department of Radiation Oncology Taixing People's Hospital Tai Xing Jiangsu Province People's Republic of China

**Keywords:** esophageal squamous cell carcinoma, Inflammatory markers, prognostic nutritional index, Radiomics

## Abstract

**Background:**

This study explores the significance of computed tomography (CT) radiomic features, along with inflammation and nutrition biomarkers, in the prognosis of postoperative patients with T3N0M0 esophageal squamous cell carcinoma (ESCC). The study aims to construct a related nomogram.

**Methods:**

A total of 114 patients were enrolled and randomly assigned to training and validation cohorts in a 7:3 ratio. Radiomic features were extracted from their preoperative chest‐enhanced CT arterial images of the primary tumor, and inflammatory and nutritional indices, including neutrophil‐to‐lymphocyte ratio (NLR), lymphocyte‐to‐monocyte ratio (LMR), platelet‐to‐lymphocyte ratio (PLR), systemic immune‐inflammation index (SII), and prognostic nutritional index (PNI), were calculated based on laboratory data from the 3 days before surgery. Intra‐class correlations coefficient (ICC) and least absolute shrinkage and selection operator (Lasso) were applied to screen valuable radiomics features predicting overall survival (OS), and the Rad‐score was calculated. In the training cohort, univariate and multivariate Cox regression analyses identified independent prognostic factors, which were adopted to establish the nomogram.

**Results:**

Eight radiomic features were selected for Rad‐score calculation. Multivariate Cox regression revealed Rad‐score, PNI, NLR, and PLR as independent prognostic factors for ESCC patients (*p* < 0.05). A nomogram was constructed based on these variables. The concordance index (C‐index) for the nomogram was 0.797 (95% CI: 0.726–0.868) in the training cohort and 0.796 (95% CI: 0.702–0.890) in the validation cohort. Calibration curves indicated good calibration ability, and the receiver operating characteristic (ROC) analysis demonstrated superior discriminative ability for the nomogram in comparison to the Rad‐score alone. Decision curve analysis (DCA) confirmed the clinical utility of the nomogram.

**Conclusion:**

We developed and validated a nomogram for predicting the OS of postoperative T3N0M0 ESCC patients, integrating nutritional, inflammatory markers, and radiomic signature. The combined nomogram can serve as a robust tool for risk stratification and clinical management.

## 1 INTRODUCTION

Esophageal cancer (EC) poses a significant threat to human health as a digestive tract tumor. In 2018, there were an estimated 572 034 new EC cases globally, ranking it seventh in incidence and sixth in mortality. China stands out as the country with the highest EC incidence and mortality rates worldwide, accounting for approximately 54.1% of new cases, among these cases, 90% are esophageal squamous cell carcinoma (ESCC), which differs from the predominant adenocarcinoma in Western countries.^1^, ^2^, ^3^ The tumor‐node‐metastasis (TNM) staging system has gained widespread recognition and application in prognostic analysis. However, advancements in treatment modalities have led to varying prognoses even among patients with the same TNM stage.^4^ Consequently, the urgent need to identify prognostic factors that facilitate risk classification and clinical management has become evident.

According to the guidelines of the National Comprehensive Cancer Network (NCCN), neoadjuvant chemoradiotherapy followed by surgery has become the preferred treatment option for patients with T3N0M0 stage esophageal cancer. Despite the recommendation by surgical oncologists for neoadjuvant therapy, traditional beliefs in China make surgery a more readily accepted primary treatment method for many patients. However, due to the lack of high‐quality randomized data, there has been ongoing debate regarding the therapeutic benefits of adjuvant treatment after esophageal cancer surgery. Moreover, the wide spectrum of survival times that exists even after complete resection of the same‐staged esophageal cancer demonstrates the imperative need for personalized therapy. For patients in this category, it is particularly crucial to identify high‐risk individuals based on clinical indicators and provide tailored, individualized treatments for them.

Radiomics is a technique that involves the in‐depth analysis of first‐order, second‐order, and high‐order texture features on medical images, reflecting the pathological and physiological information as well as the internal heterogeneity of tumors. It provides more detailed information compared to traditional clinical tumor staging and is utilized in tumor diagnosis, efficacy assessment, and prognosis prediction.^5^, ^6^ Previous studies have shown that radiomics models have significant predictive value in the accurate preoperative staging of resectable esophageal cancer,^7^ evaluating the effectiveness of neoadjuvant therapy,^8^ and assessing postoperative survival prognosis.^9^ Additionally, mounting evidence suggests that systemic inflammation and nutritional status play roles in the occurrence and development of tumors, affecting clinical outcomes. Key inflammation‐based scores such as neutrophil‐to‐lymphocyte ratio (NLR), lymphocyte‐to‐monocyte ratio (LMR), platelet‐to‐lymphocyte ratio (PLR), systemic immune‐inflammation index (SII), and prognostic nutritional index (PNI) based on serum albumin and total lymphocyte count are widely used in tumor prognosis assessment.^10^, ^11^ In this study, we aim to explore the relationship between radiomic features and inflammation‐ and nutrition‐based biomarkers with the prognosis of postoperative T3N0M0 ESCC patients. Based on the identification of factors influencing patient prognosis, we intend to construct a predictive nomogram, providing valuable insights for patient prognosis assessment and prediction.

## 2 MATERIALS AND METHODS

Ethical approval by the Institutional Ethics Review Committee was obtained for this retrospective study, and the requirement to obtain informed consent was waived. The study was approved by the medical research ethics committee of Taixing People's Hospital (XJS2023060) and was performed in accordance with the Declaration of Helsinki.

### 2.1 Patients

The study retrospectively analyzed the demographic data of 114 patients who underwent radical esophagectomy at Taixing People's Hospital from January 2010 to December 2017. Inclusion criteria were as follows: (1) Postoperative pathology confirmed ESCC staged as T3N0M0 according to the 7th edition of the American Joint Committee on Cancer (AJCC) staging system. (2) No clear contraindications for radiotherapy and chemotherapy. (3) Preoperative blood routine examination should be conducted within three days. (4) A standard computed tomography (CT) scan performed before surgery within 2 weeks. (5) Patients with detailed clinicopathological data. Exclusion criteria included: (1) Patients who received neoadjuvant therapy. (2) The lesion was too small to be recognized by CT. (3) Patients with any missing clinical, laboratory, or follow‐up information. (4) Non‐primary tumors. (5) Survival time less than 1 month. The study collected clinical and pathological parameters such as age, gender, smoking, alcohol consumption, vascular involvement, neural involvement, tumor differentiation, maximum tumor diameter, postoperative adjuvant therapy, NLR, LMR, PLR, SII, and PNI. NLR, LMR, PLR, SII, and PNI were calculated using specific formulas: NLR = neutrophil counts/lymphocyte counts, LMR = lymphocyte count/monocyte count, PLR = absolute platelet count/lymphocyte count, SII = platelet counts × neutrophil counts/lymphocyte counts, PNI = serum albumin level (g/L) + 5 × absolute lymphocyte count (mm^3^). Nutritional and inflammatory indices were derived from blood samples collected 3 days before surgery. Overall survival (OS) was defined as the time from diagnosis to death from any cause or the end of follow‐up, with all patients having a follow‐up duration exceeding 5 years until their demise.

### 2.2 Follow‐up

The end point of this study was OS, which is defined as the interval from the first day of pathological diagnosis to the date of all‐cancer death or the date of the last follow‐up. The patients were followed up with regular checks in the outpatient department, such as physical examination, blood routine, and the CT scan of the chest. Patients were postoperatively followed up with chest CT every 3 to 6 months for the initial 2 years and then the patients were followed up every 6 months from the 3rd to 5th year. The deadline date of follow‐up was December 31, 2022.

### 2.3 Data and images collection

All patients, who underwent enhanced CT scans in the CT room of Taixing People's Hospital from January 2010 to December 2017, were included in the study. The CT scans were performed using a 64‐slice LightSpeed VCT scanner (General Electric, Milwaukee, Wisconsin, USA). The specific scan parameters were as follows: tube voltage of 120 kV, tube current of 160 mAs, rotation time of 0.4 s or 0.5 s, and detector collimation of 64 × 0.625 mm or 64 × 1.25 mm. The scanning field of view was 350 mm, and the pixel size was 512 × 512, with a slice thickness of 5 mm. Standard supine position‐enhanced scans were performed, and 85 mL of iodinated contrast agent (iodixanol injection, Yangtze River Pharmaceutical Group, Jiangsu, China) was injected through the cubital vein at a rate of 3.0 mL/s using a power injector. Arterial images were collected 35 s after the injection delay. All CT images were reconstructed using standard kernels and stored in the Picture Archiving Communication System (PACS) through the hospital's local area network. Patient‐specific enhanced CT images and relevant data were retrieved from the PACS system using USB drives or CDs and subsequently imported into the radiomic analysis software for further processing.

### 2.4 Image segmentation

The 3D‐slicer software (version 4.13.0, https://www.slicer.org/) is an open‐source and free platform designed for biomedical research, specifically for processing medical imaging data. It enables the importation of enhanced CT images of patients within the scope of the study. Using specific modules within the software, regions of interest (ROIs), such as tumor primary lesions, are delineated meticulously on the CT images by deputy directors and above, who possess over a decade of experience in delineating target areas for esophageal cancer radiotherapy. The parameters for ROI were adjusted to the same window width and level (window width of 500 HU and window level of 40 HU). According to the CT diagnostic criteria of esophageal cancer lesions, an area with a thickness of the esophageal wall ≥5 mm or lumen occlusion diameter > 10  mm was regarded as the primary tumor area. Intraluminal gas, oral contrast agent, and adjacent normal structures were excluded in ROI.

### 2.5 Radiomics feature extraction

One of the extensions within the 3D‐slicer software is the Pyradiomics plugin, an open‐source python package widely utilized for extracting texture features from CT images. In this study, a total of 107 fundamental texture features were included. This set comprised 14 morphological features, 18 first‐order features, and second‐order features, as well as high‐order features, including 24 spatial gray level co‐occurrence matrix (GLCM) features, 16 gray level run length matrix (GLRLM) features, 5 neighborhood gray‐tone difference matrix (NGTDM) features, 16 gray level size zone matrix (GLSZM) features, and 14 gray‐level dependence matrix (GLDM) features. Wavelet Transform was applied to first and second‐order features, along with high‐order features, resulting in eight categories with a total of 744 indicators. Additionally, Gaussian‐type Laplacian (LoG) filtering was performed with sigma values of 0.5, 1.0, 1.5, 2.0, and 2.5, generating 465 indicators. Consequently, each delineated ROI yielded a comprehensive total of 1316 radiomic feature indicators.

### 2.6 Radiomic feature selection and radiomics model establishment

Patients were randomly allocated to training and validation groups in a 7:3 ratio. The Pearson chi‐square test and independent sample *t*‐test were employed to analyze differences in the distribution of categorical and continuous variables between the training and validation cohorts, respectively. In the training cohort, inter‐rater reliability for quantitative features extracted from images, as determined by the intra‐class correlations coefficient (ICC), was tested among two physicians with 10 years of experience in esophageal cancer radiotherapy target delineation. Features with ICC > 0.9 were selected. Least absolute shrinkage and selection operator (Lasso) regression was then used to further refine the radiomic features, employing 10‐fold cross‐validation to prevent overfitting. Finally, a radiomic score (Rad‐score) was computed for each patient through a linear combination of selected features weighted by their respective LASSO regression coefficients.

Rad‐score was calculated by using the following formula: Radscore = *β*1 × 1+*β*2 × 2+…*βnXn*, where rad‐score was the radiomic signature and *βn* was the coefficient.

X‐tile software (Yale University, version 3.6.1) was employed to determine optimal cutoff values for continuous variables, which were subsequently transformed into categorical variables. Prognostic variables were analyzed using univariate Cox regression. Significant variables (*p* < 0.05) from the univariate analysis were incorporated into the multivariate Cox regression to identify independent prognostic factors. A nomogram was constructed based on these independent prognostic factors. Discrimination of the nomogram was evaluated using receiver operating characteristic (ROC) analysis and concordance index (C‐index) assessment. Calibration curves were generated to confirm the accuracy of the nomogram. The clinical utility of the nomogram was assessed through decision curve analysis (DCA). To mitigate overfitting bias, Bootstrap analysis was performed with 1000 re‐samplings.

### 2.7 Statistical Analysis

All analyses were conducted using R statistical software (version 4.2.1, http://www.R‐project.org). The “tableone” and “rio” packages were used for *t*‐tests, chi‐square tests, and data output. “corrplot” and “glmnet” packages were utilized for lasso regression. “psych” package was employed for ICC analysis. “plyr”, “rms”, “epiDisplay”, “gtsummary”, ′survival″, “forestplot”, and ′survminer″ packages were used for univariate and multivariate Cox regression, nomogram construction, and C‐index calculation. ′rms″ and “survival” packages were used for calibration curve construction. “riskRegression” and “survival” packages were used for ROC curve construction. “rms”, “ggDCA”, “ggprism”, and “survival” packages were employed for clinical decision analysis. Statistical significance was set at *p* < 0.05.

## 3 RESULTS

### 3.1 Patient characteristics

A total of 114 patients were included in the analysis, with 79 patients in the training cohort and 35 patients in the validation cohort. Across the entire cohort, 78 (68.42%) patients were below the age of 69, and 80 (70.18%) were male. Among these patients, 52 (45.61%) had a history of alcohol consumption, and 49 (42.98%) were smokers. Additionally, 15 (13.16%) patients exhibited vascular invasion, while 16 (14.04%) had neural invasion. Of the total, 44 (38.60%) patients underwent adjuvant therapy. Furthermore, based on the X‐tile analysis, patients were stratified into groups: high NLR (NLR > 4.22), low NLR (NLR ≤ 4.22), high LMR (LMR > 2.18), low LMR (LMR ≤ 2.18), high PLR (PLR > 194.12), low PLR (PLR ≤ 194.12), high SII (SII > 712.74), low SII (SII ≤ 712.74), high PNI (PNI > 44.90), and low PNI (PNI ≤ 44.90). The median follow‐up duration for the entire study cohort was 60 months (range: 1−99 months). During the follow‐up period, there were 55 recorded deaths, with survival rates at 2 years, 3 years, and 5 years being 74.56%, 62.28%, and 51.75%, respectively. No statistically significant differences in clinical characteristics were observed between the training and validation cohorts; detailed information is provided in Table 1.

**TABLE 1:** Characteristics of the training and validation cohorts.

		Whole cohort	Training cohort	Validation cohort	
Variables		*n* = 114	*n* = 79	*n* = 35	*p*
**Age (years)**	>69	36 (31.58)	27 (34.18)	9 (25.71)	0.498
	≤69	78 (68.42)	52 (65.82)	26 (74.29)	
**Sex**	Female	34 (29.82)	27 (34.18)	7 (20.00)	0.192
	Male	80 (70.18)	52 (65.82)	28 (80.00)	
**Smoke**	No	65 (57.02)	46 (58.23)	19 (54.29)	0.852
	Yes	49 (42.98)	33 (41.77)	16 (45.71)	
**Drink**	No	62 (54.39)	45 (56.96)	17 (48.57)	0.531
	Yes	52 (45.61)	34 (43.04)	18 (51.43)	
**Vessel**	No	99 (86.84)	69 (87.34)	30 (85.71)	1.000
	Yes	15 (13.16)	10 (12.66)	5 (14.29)	
**Nerve**	No	98 (85.96)	65 (82.28)	33 (94.29)	0.158
	Yes	16 (14.04)	14 (17.72)	2 (5.71)	
**Length (cm)**	>5.5	18 (15.79)	13 (16.46)	5 (14.29)	0.988
	≤5.5	96 (84.21)	66 (83.54)	30 (85.71)	
**Adjuvant therapy**	No	70 (61.40)	47 (59.49)	23 (65.71)	0.674
	Yes	44 (38.60)	32 (40.51)	12 (34.29)	
**NLR**	>4.22	15 (13.16)	12 (15.19)	3 (8.57)	0.507
	≤4.22	99 (86.84)	67 (84.81)	32 (91.43)	
**LMR**	>2.18	97 (85.09)	67 (84.81)	30 (85.71)	1.000
	≤2.18	17 (14.91)	12 (15.19)	5 (14.29)	
**PLR**	>194.12	12 (10.53)	9 (11.39)	3 (8.57)	0.903
	≤194.12	102 (89.47)	70 (88.61)	32 (91.43)	
**SII**	>712.74	20 (17.54)	12 (15.19)	8 (22.86)	0.468
	≤712.74	94 (82.46)	67 (84.81)	27 (77.14)	
**PNI**	>44.90	99 (86.84)	70 (88.61)	29 (82.86)	0.591
	≤44.90	15 (13.16)	9 (11.39)	6 (17.14)	
**Rad‐score (mean (± SD))**		−2.255 (**± **0.468)	−2.278 (**± **0.367)	−2.202 (**± **0.644)	0.430

Abbreviations: LMR, lymphocyte‐to‐monocyte ratio; NLR, neutrophil‐to‐lymphocyte ratio; PLR, platelet‐to‐lymphocyte ratio; PNI, prognostic nutritional index; Rad score, radiomic score; SD, standard deviation; SII: systemic immune‐inflammation index.

### 3.2 Feature selection and model construction in radiomics

In this study, a total of 1316 radiomic features were initially considered. After conducting the ICC test and excluding features with coefficients less than 0.9, 625 features remained for further analysis. Dimensionality reduction and feature selection were performed using Lasso‐Cox regression. The lambda (*λ*) value associated with the least bias was selected, and features with non‐zero coefficients were retained. Ultimately, eight radiomic features were identified to construct the radiomics prediction model, as detailed in Table 2. The radiomics prediction model's score, referred to as Rad‐score, was calculated using the following formula: Rad‐score = −1.985885254*original‐shape‐Sphericity

**TABLE 2:** Eight selected radiomics features.

Radiomic features	Coefficients of LASSO‐Cox
original‐shape‐Sphericity	−1.985885254
wavelet‐LHH‐firstorder‐Minimum	−0.000490631
wavelet‐LHH‐gldm‐LargeDependenceHighGrayLevelEmphasis	5.163E‐05
wavelet‐LHH‐glszm‐LargeAreaHighGrayLevelEmphasis	2.94148E‐09
wavelet‐HHH‐firstorder‐Kurtosis	0.000291409
log‐sigma‐0‐5‐mm‐3D‐glcm‐Id	1.541401391
log‐sigma‐0‐5‐mm‐3D‐glcm‐InverseVariance	−3.506788668
log‐sigma‐2‐5‐mm‐3D‐gldm‐DependenceEntropy	−0.185677568

Abbreviation: LASSO, least absolute shrinkage and selection operator.

−0.000490631*wavelet‐LHH‐firstorder‐Minimum

+5.163E‐05*wavelet‐LHH‐gldm‐LargeDependenceHighGrayLevelEmphasis

+2.94148E‐09*wavelet‐LHH‐glszm‐LargeAreaHighGrayLevelEmphasis

+0.000291409*wavelet‐HHH‐firstorder‐Kurtosis

+1.541401391*log‐sigma‐0‐5‐mm‐3D‐glcm‐Id

−3.506788668*log‐sigma‐0‐5‐mm‐3D‐glcm‐InverseVariance

−0.185677568*log‐sigma‐2‐5‐mm‐3D‐gldm‐DependenceEntropy。

### 3.3 Univariate and multivariate Cox analysis

Univariate COX analysis revealed significant correlations between tumor length (*p* < 0.001), adjuvant therapy (*p* = 0.040), NLR (*p* < 0.001), LMR (*p* < 0.001), PLR (*p* = 0.002), SII (*p* = 0.008), PNI (*p* < 0.001), Radscore (*p* < 0.001), and OS. These factors were incorporated into the multivariate Cox regression model. In the multivariate analysis, NLR (*p* < 0.001), PLR (*p* = 0.035), PNI (*p* = 0.014), and Radscore (*p* = 0.028) were identified as independent prognostic factors (Table 3).

**TABLE 3:** Univariate analysis and multivariate COX analysis.

		Univariable		Multivariable	
Variables		HR (95% CI)	*p*	HR (95% CI)	*p*
Age (years)	>69	Reference			
	≤69	0.571 (0.300‐1.089)	0.089		
Sex	Female	Reference			
	Male	0.845 (0.437‐1.634)	0.617		
Smoke	No	Reference			
	Yes	1.143 (0.6‐2.177)	0.684		
Drink	No	Reference			
	Yes	1.197 (0.631‐2.269)	0.582		
Vessel	No	Reference			
	Yes	0.806 (0.286‐2.272)	0.684		
Nerve	No	Reference			
	Yes	1.122 (0.494‐2.549)	0.783		
Length (cm)	>5.5	Reference		Reference	
	≤5.5	0.264 (0.129‐0.539)	<0.001	2.070 (0.643‐6.661)	0.223
Adjuvant therapy	No	Reference		Reference	
	Yes	0.479 (0.237‐0.966)	0.040	0.593 (0.237‐1.483)	0.264
NLR	>4.22	Reference		Reference	
	≤4.22	0.136 (0.066‐0.279)	<0.001	0.115 (0.031‐0.431)	0.001
LMR	>2.18	Reference		Reference	
	≤2.18	5.760 (2.793‐11.875)	<0.001	2.285 (0.794‐6.579)	0.126
PLR	>194.12	Reference		Reference	
	≤194.12	0.276 (0.121‐0.631)	0.002	0.316 (0.108‐0.921)	0.035
SII	>712.74	Reference		Reference	
	≤712.74	0.362 (0.17‐0.769)	0.008	2.913 (0.754‐11.254)	0.121
PNI	>44.90	Reference		Reference	
	≤44.90	5.39 (2.419‐12.012)	<0.001	4.070 (1.336‐12.401)	0.014
Rad‐score		9.959 (4.325‐22.933)	<0.001	4.801 (1.190‐19.376)	0.028

Abbreviations: CI, confidence interval; HR, hazard ratio; LMR, lymphocyte‐to‐monocyte ratio; NLR, neutrophil‐to‐lymphocyte ratio; PLR, platelet‐to‐lymphocyte ratio; PNI, prognostic nutritional index; Rad score, radiomic score; SD, standard deviation; SII, systemic immune‐inflammation index.

### 3.4 Establishment and validation of prognostic nomogram and risk stratification

**FIGURE 1:**
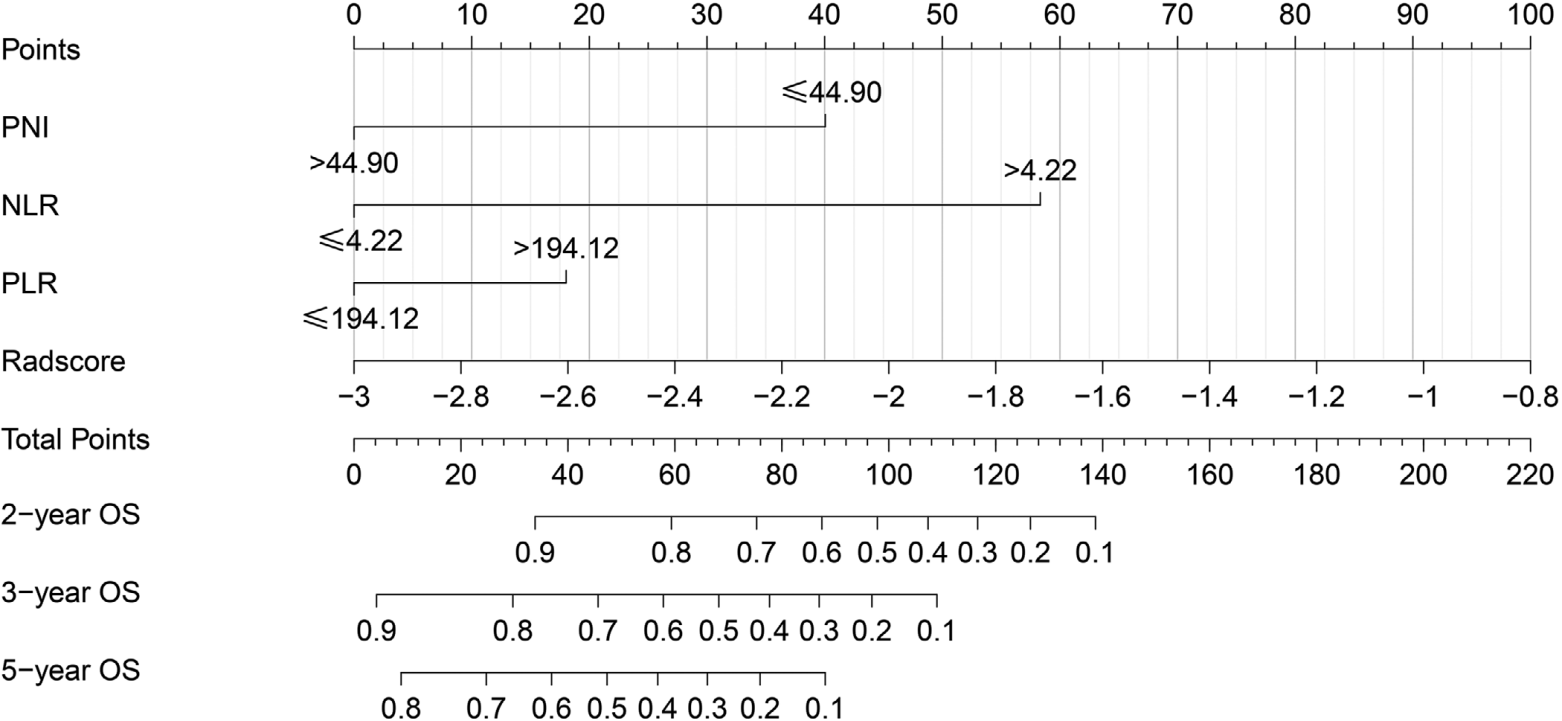
Nomogram for predicting 2‐, 3‐ and 5‐year overall survival of patients with esophageal cancer.

**FIGURE 2:**
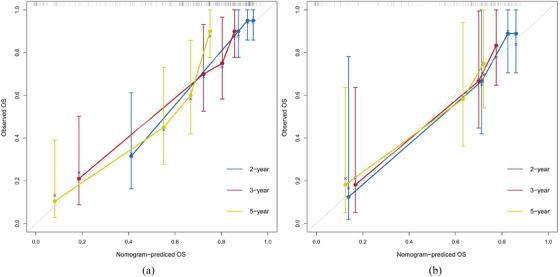
Calibration curves of the nomogram. (a) Calibration curves of 2‐, 3‐ and 5‐year overall survival in the training cohort; (b) Calibration curves of 2‐, 3‐ and 5‐yr overall survival in the validation cohort.

**FIGURE 3:**
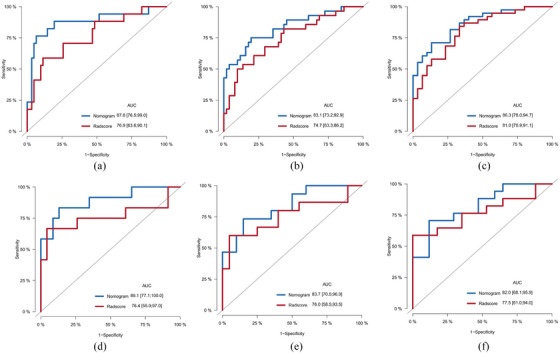
Receiver operating characteristic curves of the nomogram versus Rad‐score. (a, b, c) For 2‐, 3‐ and 5‐year overall survival in the training cohort; (d, e, f) for 2‐, 3‐ and 5‐year overall survival in the validation cohort. Rad‐score, radiomic score.

**FIGURE 4:**
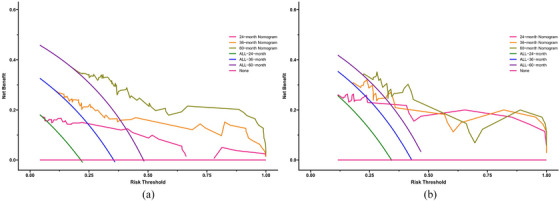
Decision curve analysis for survival prediction. (a) For 2‐, 3‐ and 5‐year overall survival in the training cohort; (b) for 2‐, 3‐ and 5‐year overall survival in the validation cohort.

**FIGURE 5:**
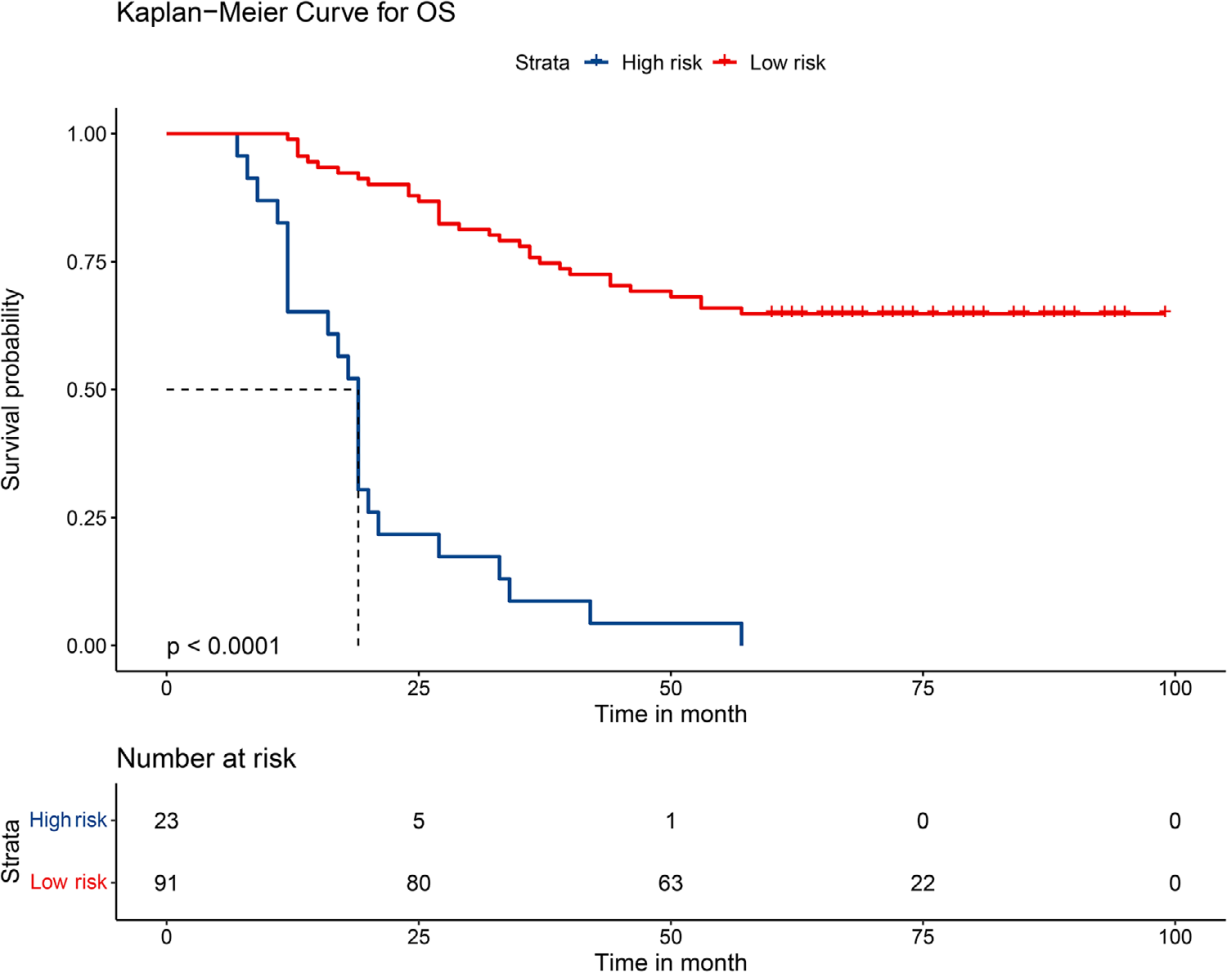
Kaplan–Meier method estimate of overall survival in the whole cohort. Low risk refers to a total score of ≤64.74, high risk refers to a total score of > 64.74.

The four identified independent prognostic factors were utilized to create a nomogram (Figure 1). In the training and validation cohorts, the nomogram demonstrated a c‐index of 0.797 (95% CI: 0.726−0.868) and 0.796 (95% CI: 0.702−0.890), respectively. Calibration curves for 2‐year, 3‐year, and 5‐year survival rates were plotted to assess its calibration ability (Figure 2). The nomogram's predictions closely matched the actual survival rates. ROC curves indicated superior predictive performance of the nomogram over the 2‐year, 3‐year, and 5‐year periods Figure 3. In the training cohort, the nomogram achieved area under the curve (AUC) values of 0.878 (95% CI: 0.765−0.990), 0.831 (95% CI: 0.732−0.929), and 0.863 (95% CI: 0.780−0.947) for predicting 2‐year, 3‐year, and 5‐year OS, respectively. In the validation cohort, corresponding AUC values were 0.859 (95% CI: 0.729−0.988), 0.837 (95% CI: 0.705−0.969), and 0.820 (95% CI: 0.681−0.959), indicating excellent discriminative ability of the nomogram. Additionally, the DCA further indicated the nomogram's good clinical applicability in predicting 2‐year, 3‐year, and 5‐year survival rates Figure 4. Furthermore, using X‐tile analysis, the optimal cutoff value for the nomogram's risk score was determined, and log‐rank tests validated the survival differences between high (> 64.74) and low (≤64.74) risk groups (*p* < 0.001), detailed information is provided in Figure 5. Detailed results can be found in Table S1.

## 4 DISCUSSION

Utilizing radiomics and inflammatory nutritional biomarkers, our research has developed and validated a nomogram prediction model for OS in esophageal cancer, characterized by high discriminative ability and robustness.

Previous studies have analyzed the relationship between radiomic features and prognosis in EC patients. For instance, Gong et al.^12^ developed a hybrid radiomic nomogram by integrating radiomic features, deep learning characteristics, T stage, and clinical factors such as concurrent chemoradiotherapy. This model was designed to predict local recurrence‐free survival (LRFS) in patients with ESCC undergoing definitive (chemo)radiotherapy across multiple centers. The C‐index for predicting LRFS using this model was 0.82 in the training cohort, 0.78 in the internal validation cohort, and 0.76 in the external validation cohort, respectively. Xie et al.^13^ extracted seven sub‐regional radiomic features based on the Hounsfield unit (HU) values and local entropy values from CT images of tumor regions in patients to predict the 3‐year survival rate after concurrent chemoradiotherapy for EC. The model achieved a C‐index of 0.729 and an AUC of 0.811 in the training set. Similarly, Zeng et al.^14^ analyzed 154 EC patients for OS and progression‐free survival (PFS) following concurrent chemoradiotherapy. Their final radiomic model included 11 enhanced CT image features for OS and 8 for disease‐free survival (DFS), with C‐index for predicting OS and DFS being 0.730 and 0.685 in the training cohort, and 0.672 and 0.666 in the validation cohort, respectively. Our research supports this conclusion. This study was based on 114 patients and 1316 radiomic features. Through a rigorous selection process, eight radiomics features highly correlated with prognosis were selected by LASSO with 10‐fold cross‐validation, including one shape‐based feature, four wavelet features, and three Gaussian‐filtered features. The OS prediction model combined with eight sub‐regional radiomics features was constructed with a C‐index of 0.73 in the training cohort.

The extraction and selection of radiomic features represent crucial steps in radiomic studies. In our research, to enable multi‐resolution analysis of images and visualize different levels of image details, we not only extracted primary shape features, histogram features (first‐order features), and texture features but also transformed and combined the original images through wavelet filtering and Gaussian filtering. Identified features in this study encompassed one shape‐based feature, four wavelet features, and three Gaussian‐filtered features. The predominant inclusion of wavelet and Gaussian‐filtered features in the selected radiomic features for model construction may be attributed to the fact that wavelet transformation feature parameters reflect multi‐frequency information at different scales, which is imperceptible to the naked eye and thus cannot be quantified in tumor heterogeneity. Similarly, LoG feature parameters can extract texture features with significant discriminative power from multiple spatial scales, effectively removing noise and smoothing images, thereby enhancing the efficiency of capturing phenotype features associated with tumor heterogeneity.^15^, ^16^

The fundamental assumption of radiomics is that quantitative analysis of imaging data provides valuable diagnostic, prognostic, or predictive information. By assessing the heterogeneity of tumor imaging, we can evaluate the heterogeneity of genomic expression and patient prognosis. Greater genomic heterogeneity is associated with an increased likelihood of drug resistance and metastasis. There is substantial evidence suggesting that quantitative imaging biomarkers associated with tumor heterogeneity may offer crucial information for predicting treatment response and prognosis in EC patients undergoing chemoradiotherapy.^17^, ^18^, ^19^, ^20^, ^21^ This study identified three parameters from Gaussian‐filtered GLCM transformation as effective predictors in the radiomic model. Specifically, inverse difference (measuring the local homogeneity of the image), difference variance (another measure of heterogeneity, assigning higher weight to deviations of different intensity levels from the mean), and dependence entropy (gray‐level dependent entropy) serve as indicators of texture homogeneity or heterogeneity. GLCM entropy reflects local irregularities within the image; higher entropy values indicate greater image complexity. Conversely, inverse variance reflects the magnitude of local variations in image texture. If different parts of the texture are more uniform and change slowly, the inverse variance will be larger. Most of the imaging features we selected are indicators associated with the heterogeneity of image texture, indirectly suggesting that tumor heterogeneity is more correlated with treatment outcomes and survival prognosis than tumor volume or shape. This parallels many other prediction models based on CT imaging radiographs.^13^, ^14^, ^17^, ^22^, ^23^, ^24^

Most tumors result from the interplay of environmental factors and genetic alterations, reflecting the synergistic effects of multiple factors. Therefore, it is challenging to assess the comprehensive postoperative outcomes of individual cancer patients without modeling any powerful risk factors.^25^ Nomograms based on multi‐factorial regression analysis are indispensable. Integrating radiomic features with conventional staging systems and other clinical‐pathological risk factors can optimize the performance of prediction models.^24^ Chu et al.^22^ demonstrated that a combined predictive model integrating radiomics based on MRI and clinical risk factors provided superior prognostic efficacy for DFS and OS in patients with ESCC compared to a standalone radiomic model. Similarly, Huang et al.^26^ showed that a combined radiomic‐clinico‐pathological nomogram estimated DFS with a C‐index of 0.72 (95% CI: 0.71, 0.73), outperforming a standalone clinicopathological nomogram (C‐index: 0.691; 95% CI: 0.68, 0.70). Moreover, it improved classification accuracy (net reclassification improvement: 0.182; 95% CI: 0.02, 0.31; p = 0.02). Cui et al.^23^ incorporated radiomic features into a nomogram alongside changes in the homologous recombination repair pathway to predict progression‐free survival in patients with ESCC. The integrated model combining radiomics and genomics achieved higher C‐index in both the training cohort (0.616 vs. 0.587 or 0.557) and the validation cohort (0.649 vs. 0.625 or 0.586) compared to either the radiomics or genomics models alone. Cui et al.’s study integrated genomic features that are typically challenging to obtain, facilitating their clinical applicability. Therefore, in addition to radiomic features, we incorporated easily accessible nutritional and inflammatory markers into our model, which are highly correlated with prognosis in patients with EC.

In 1863, the relationship between inflammation and cancer was first revealed by Rudolf Virchow.^27^ Virchow hypothesized that cancer origin is related to chronic inflammation. Subsequent studies elucidated the connection between inflammation and tumors,^28^, ^29^ suggesting that inflammation promotes the onset, progression, malignant transformation, invasion, and distant metastasis of tumors, closely linked to patient prognosis. NLR, along with PLR and other indicators, is the most commonly used marker to assess systemic inflammation in cancer patients. Numerous studies have confirmed its role in prognostic prediction for various cancers. Ishibashi et al. further consolidated the strong correlation between poor prognosis in EC patients and NLR values through a meta‐analysis. Deng et al.^31^ demonstrated a significant association between elevated PLR and poor prognosis in EC, with high PLR closely related to worse clinicopathological features in EC patients.  Feng et al.^32^ demonstrated preoperative NLR and PLR were significant predictors of OS in patients with ESCC. PNI is an immune nutritional status evaluation index that integrates lymphocyte count and serum albumin, and it has higher sensitivity compared to individual markers. It is easily accessible, cost‐effective, and has good predictive performance. Li et al.^33^ reviewed nine observational studies, indicating a significant correlation between low PNI scores and poorer OS in EC and recurrence‐free survival rates in ESCC. Jiang et al.^11^ conducted a meta‐analysis of 72 studies, involving 22,260 patients, confirming the association between decreased PNI and poorer OS in EC. Zhang et al.^34^ demonstrated that the preoperative high SII and low PNI are powerful indicators of aggressive biology and poor prognosis for patients with ESCC. The combination of SII and PNI can enhance the accuracy of prognosis. However, our research suggests that SII is not related to the prognosis of T3N0, which may be related to different inclusion criteria.

In our study, multi‐factorial Cox regression confirmed that both NLR and PLR are independent prognostic factors for ESCC patients, consistent with previous research. Moreover, we used X‐tile software to select the optimal cutoff values based on patients' OS as the study endpoint, greatly enhancing the model's accuracy. In our study, we demonstrated that a combined radiomic‐clinical nomogram outperformed a standalone radiomic nomogram in terms of prognostic performance, exhibiting higher C‐index and better calibration. In the training cohort, the C‐index for the radiomics‐only model was 0.730 (95% CI: 0.646−0.814). Upon inclusion of nutritional and inflammatory features, the C‐index of this model increased to 0.797 (95% CI: 0.726−0.868). Additionally, the C‐index of this combined model surpassed that reported by Cui et al (0.797 vs. 0.619). While external validation of the proposed nomogram is lacking, decision curve analysis indicated the superiority of the integrated nomogram over the radiomics‐only model, suggesting the incremental value of nutritional and inflammatory features in enhancing personalized prognostic prediction based on radiomic features.

Unlike previous studies that primarily analyzed prognostic investigations of patients at all disease stages, our current research focuses solely on patients with T3N0 stage ESCC. Currently, based on the results of cross‐trials and the NEOCRTEC5010 trial, neoadjuvant chemoradiotherapy combined with surgery is the standard regimen for locally advanced resectable ESCC.^35^, ^36^ However, pretreatment staging of T3N0 patients is prone to underestimation, and some patients are unwilling to undergo neoadjuvant treatment, while the concept of neoadjuvant therapy among surgeons in primary hospitals is relatively weak. For these reasons, surgery remains the primary treatment modality for T3N0M0 ESCC patients in the real world. Additionally, due to the lack of high‐quality prospective studies,^37^, ^38^, ^39^ the benefits of adjuvant therapy for these patients have been a contentious topic. For patients diagnosed with pathologic T3N0M0 ESCC, the widely accepted TNM staging system does not provide additional value. Therefore, identifying high‐risk individuals based on clinical indicators and providing appropriate personalized treatment is crucial. According to the 8th edition of the cancer staging manual, T3N0M0 patients are classified into stage IIA and stage IIB. There is a difference in OS between clinical stages IIA and IIB, indicating that there is heterogeneity in survival outcomes even within the same clinical stage (Stage II). In our study, radiomic features successfully identified high‐risk patients with poorer survival outcomes, indicating that these patients may require more aggressive or intensified postoperative treatment.

Although this study established a reliable nomogram, there are still some limitations. Firstly, it is a single‐center retrospective study that only included patients who underwent surgery and were staged as T3N0M0, leading to potential selection bias and potentially unreliable and non‐reproducible results. To further validate, the value of CT radiomics in predicting OS in ESCC patients needs to be further confirmed in prospective, multi‐center, and large‐scale studies. Secondly, all ROIs were manually delineated, which is time‐consuming and subject to inter‐observer variability. Therefore, future exploration of an automatic segmentation method is warranted to save time and reduce errors. Thirdly, this study only delineated target regions on arterial phase CT images, without incorporating features from venous phase and equilibrium phase images, potentially overlooking more meaningful parameters.

## 5 CONCLUSION

We developed and validated a nomogram for predicting the OS of postoperative T3N0M0 ESCC patients, integrating nutritional, inflammatory markers, and radiomic features. The combined nomogram can serve as a robust tool for risk stratification and clinical management.

## AUTHOR CONTRIBUTIONS

Hui Ma, Hongxun Ye, and Songbing Qin designed the study. Hui Ma prepared figures and wrote the manuscript. Hui Ma, Yangchen Liu, Hongxun Ye and Fei Gao collected the follow‐up data. Hui Ma performed the statistical analysis. All authors reviewed and approved the final manuscript.

## CONFLICT OF INTEREST STATEMENT

The authors declare no conflicts of interest.

## ETHICS APPROVAL

The study was approved by the ethics committee of Taixing People's Hospital (XJS2023060) and was performed in accordance with the standards of the Declaration of Helsinki. Written informed consent was obtained from all participants in the study. The manuscript has not been published elsewhere.

## Supporting information



Supporting Information

## Data Availability

The data that support the findings of this study are available from the univariate and multivariate Cox analyzing author upon reasonable request.
