# Supplementary material for: A prognostic nomogram for T3N0M0 esophageal squamous cell carcinoma patients undergoing radical surgery based on computed tomography radiomics and inflammatory nutritional biomarkers
Source: J Appl Clin Med Phys. 2024 Sep 6;25(11):e14504. doi: 10.1002/acm2.14504 (PMC11539971; doi:10.1002/acm2.14504)
Supplement: Supplementary file 1 — Supporting Information [file ACM2-25-e14504-s001.docx]

| Supplement Table 1 Characteristics of the High risk and low risk cohorts. | | | | | |
| --- | --- | --- | --- | --- | --- |
| Variables |  | Overall | High | Low | p |
|  |  | n=114 | n=23 | n=91 |  |
| Age (years) | ＞69 | 36 (31.58) | 11 (47.83) | 25 (27.47) | 0.104 |
|  | ≤69 | 78 (68.42) | 12 (52.17) | 26 (74.29) |  |
| Sex | Female | 34 (29.82) | 6 (26.09) | 28 (30.77) | 0.854 |
|  | Male | 80 (70.18) | 17 (73.91) | 63 (69.23) |  |
| Smoke | No | 65 (57.02) | 10 (43.48) | 55 (60.44) | 0.218 |
|  | Yes | 49 (42.98) | 13 (56.52) | 36 (39.56) |  |
| Drink | No | 62 (54.39) | 9 (39.13) | 53 (58.24) | 0.159 |
|  | Yes | 52 (45.61) | 14 (60.87) | 38 (41.76) |  |
| Vessel | No | 99 (86.84) | 19 (82.61) | 80 (87.91) | 0.744 |
|  | Yes | 15 (13.16) | 4 (17.39) | 11 (12.09) |  |
| Nerve | No | 98 (85.96) | 18 (78.26) | 80 (87.91) | 0.393 |
|  | Yes | 16 (14.04) | 5 (21.74) | 11 (12.09) |  |
| **Grade** | Well | 9 (7.89) | 4 (17.39) | 5 (5.49) | 0.082 |
|  | Moderate | 96 (84.21) | 16 (69.57) | 80 (87.91) |  |
|  | Poorly | 9 (7.89) | 3 (13.04) | 6 (6.59) |  |
| Length (mm) | ＞55 | 18 (15.79) | 10 (43.48) | 8 (8.79) | <0.001 |
|  | ≤55 | 96 (84.21) | 13 (56.52) | 83 (91.21) |  |
| Adjuvant therapy | No | 70 (61.40) | 19 (82.61) | 51 (56.04) | 0.036 |
|  | Yes | 44 (38.60) | 4 (17.39) | 40 (43.96) |  |
| NLR | ＞4.22 | 15 (13.16) | 13 (56.52) | 2 (2.20) | <0.001 |
|  | ≤4.22 | 99 (86.84) | 10 (43.48) | 89 (97.80) |  |
| LMR | ＞2.18 | 97 (85.09) | 14 (60.87) | 83 (91.21) | 0.001 |
|  | ≤2.18 | 17 (14.91) | 9 (39.13) | 8 (8.79) |  |
| PLR | ＞194.12 | 12 (10.53) | 7 (30.43) | 5 (5.49) | 0.002 |
|  | ≤194.12 | 102 (89.47) | 16 (69.57) | 86 (94.51) |  |
| SII | ＞712.74 | 20 (17.54) | 12 (52.17) | 8 (8.79) | <0.001 |
|  | ≤712.74 | 94 (82.46) | 11 (47.83) | 83 (91.21) |  |
| PNI | ＞44.90 | 99 (86.84) | 15 (65.22) | 84 (92.31) | 0.002 |
|  | ≤44.90 | 15 (13.16) | 8 (34.78) | 7 (7.69) |  |
| Radscore (mean (±SD)) |  | -2.255(±0.468) | -1.676(±0.646) | -2.401(±0.256) | <0.001 |

|  |
| --- |

NOTE: NLR: neutrophil-to-lymphocyte ratio; LMR: lymphocyte-to-monocyte ratio; PLR: platelet-to-lymphocyte ratio; SII: systemic immune‐inflammation index; PNI: prognostic nutritional index; SD: standard deviation.
